# Protein profiling of infected human gastric epithelial cells with an Iranian *Helicobacter pylori* clinical isolate 

**Published:** 2017

**Authors:** Zeinab Fazeli, Masoud Alebouyeh, Vahid Mansouri, Habib Malekpour

**Affiliations:** 1 *Gastroenterology and Liver Diseases Research Center, Research Institute for Gastroenterology and Liver Diseases, Shahid Beheshti University of Medical Sciences, Tehran, Iran*; 2 *Gastrointestinal and Liver Diseases Research Center, Iran University of Medical Sciences, Tehran, Iran*; 3 *Foodborne and Waterborne Diseases Research Center, Research Institute for Gastroenterology and Liver Diseases, Shahid Beheshti University of Medical Sciences, Tehran, Iran*; 4 *Proteomics Research Center, Shahid Beheshti University of Medical Sciences, Tehran, Iran *; 5 *Basic and Molecular Epidemiology of Gastrointestinal Disorders Research Center, Research Institute for Gastroenterology and Liver Diseases, Shahid Beheshti University of Medical Sciences, Tehran, Iran*

**Keywords:** Gastric epithelial cells, Mass spectrometry, *Helicobacter pylori*, Proteomic analysis

## Abstract

**Aim:**

The main objective of this study was to use high throughput approach to characterize the response of human gastric epithelial cells to *Helicobacter pylori* (*H. pylori*) infection at protein level.

**Background:**

Alteration of host cell protein profiles occurs due to *H.* *pylori* infection. This alteration seems to be strain specific. High throughput approaches, such as proteomics, can describe changes that occurs at the protein levelin the infected cells in response to *H.* *pylori* infection. In accordance with this point of view, we used two dimensional electrophoresis (2-DE)/MS to determine changes in proteome profile of gastric epithelial cells infected with a clinical isolate of *H. pylori* from an Iranian patient.

**Methods:**

Human gastric epithelial cells (AGS) were infected by an Iranian *H.* *pylori* isolate (complete *cag*PAI, *vacA s2m2*, *babA2*, *iceA1*, *sabA*). The altered protein patterns separated by 2-DE were identified by matrix-assisted laser desorption/ionization-time of flight mass spectrometry (MALDI-TOF MS) analysis.

**Results:**

The results showed 40 spots with significantly different intensities between the 2-DE gels. Protein SETSIP and Endoplasmic reticulum resident protein 29 were identified by MALD-TOF and Mascot search. Proteomic analysis for functional roles of these proteins showed that mechanisms to deal with stress conditions and transcriptional activator related to cell reprogramming are involved in *H*.* pylori* infection.

**Conclusion:**

Using high throughput approaches, such as proteomics, we can provide further molecular details about interaction of *H. pylori* strains with the infected cells at protein level.

## Introduction

*Helicobacter pylori,* a spiral-shaped, microaerophilic, Gram-negative bacterium, has been related to gastric or duodenal ulcer, atrophic gastritis, adenocarcinoma and mucosa-associated lymphoid tissue lymphoma(1). Epidemiological studies have demonstrated that H. *pylori* infection is present in more than 80% of developing countries and less than 40% in the developed ones(2). Iranian studies show that although the prevalence of *H. pylori *infection is highly widespread among the Iranian population, as a result of improvement in the living conditions and hygiene, in recent years it has been decreased in this country (3-7).

*H. pylori *strains virulence, host susceptibility and environmental co-factors lead to the development of disease. So, only a subset of infected individuals develops serious disease during their lifetime(8). 

*H.* *pylori* infection induces the expression of proto-oncogenesis, inflammatory cytokines, inflammatory enzymes and transcription factors in human gastric epithelial cells which are necessary steps in the development of disease(9).

Since the relationship between *H.* *pylori* infection and the incidence of gastric diseases is evident, it is essential to investigate the human responses to* H. pylori.* Accordingly, to enhance understanding of the human responses to* H. pylori *and its interaction with host, many groups have examined the response of gastric epithelial cells to *H.* *pylori* by high throughput technologies such as microarrays and proteomic (9, 10). Proteomic analysis is a valuable tool for characterizing the pathogenic mechanism of gastric diseases associated with *H.* *pylori* infection by determining the differentially expressed proteins that could be the mediators in the infected cells. The results could promote a better understanding of disease processes, develop new biomarkers for diagnosis and early detection of disease; and accelerate drug development.

Depending on the virulence factors of *H.* *pylori*, the clinical outcome of the infection may be different. Since the results of Iranian studies indicate that the prevalence of *H.* *pylori* infection is still high; it is an urgent need to understand how Iranian *H.* *Pylori* strain impact the outcome of infection. Using an Iranian *H.* *pylori* isolate could help to better knowing the pathologic mechanism of *H.* *pylori*-associated gastric disease in Iran. In the present work, we used proteomics to characterize the differentially expressed proteins induced by an Iranian *H.* *Pylori* strain in human gastric epithelial cells (AGS) which are frequently used for the studies on pathologic mechanism studies. 

## Methods

***H.*** ***pylori* strain and growth conditions**

*H.* *pylori* strain HC-113(complete *cag*-PAI, *vacAs2m2*, *babA2*, *iceA1*, *sabB*), a clinical isolate from a patient with intestinal metaplasia as described previously (11), selected from microbial collection of Research Institute for Gastroenterology and Liver Diseases in Tehran, Iran. The isolate was recovered from the stocks on Brucella agar medium supplemented with fetal calf serum (10%) (v/v), horse blood (7%), selective supplement (vancomycin 2.0 mg, polymyxin B 0.05 mg and trimethoprim 1.0 mg, Merck, Germany), and amphotericin B (3 mg/l). The plates were incubated at 37 ^o^C for 3 days in a microaerobic atmosphere (5% O2, 10% CO2, and 85% N2). The grown organism was identified as *H. pylori* by Gram staining colony morphology as well as positive oxidase, catalase and urease reactions

**AGS gastric epithelial cell co-culture**

The human gastric cancer AGS (ATCC CRL-1739TM) cell line (IBRC, Tehran, Iran) was grown in 25-cm^2^ flasks with Dulbecco’s modified Eagle’smedium (Gibco, GrandIsland, NY, USA) supplemented with 10% heat-inactivated fetal bovine serum (Gibco, Grand Island, NY, USA), 1% non-essential amino acid (Gibco, Grand Island, NY, USA), 100 U ml−1 of penicillin and 100 μg ml −1 of streptomycin (Gibco, Grand Island, NY, USA) at 37◦C in a humidified incubator (Memmert, Dusseldorf, Germany) containing 5% CO2 for 2 days to reach about 70% cell confluency before the addition of *H.* *pylori* strain. Two hours prior to infection, cells were washed with PBS (1x) and the medium was replaced with fresh, antibiotic free DMEM media. The cells were washed once with PBS and 4 mL of fresh medium was added to each flask. *H.* *pylori* was re-suspended in 0.5 mL PBS and added to AGS cells at a multiplicity of infection (MOI) of 100. After 6 hours incubation in a 5% CO2/95% air incubator, AGS cells were washed once with PBS to remove non adherent bacteria then treated with radio immuno precipitation assay buffer (RIPA BUFFER) according to the manufacturer’s instructions (Sigma, USA). Then the lysate frozen in liquid nitrogen, rapidly and stored at –70 °C for future use.

**2-DE Separation and CBB G-250 Staining**

Protein concentrations were determined by the 2-D Quant Kit according to the manufacturer’s instructions (GE Healthcare, USA). Isoelectric focusing (IEF) as the first dimension electrophoresis was carried out with 7 cm (pH 3–10NL) IPG strips at -20°C according to the manufacturer's instructions. Briefly, approximately 1 mg protein was loaded onto each gel. The strips were rehydrated in the absence of electric field for 4 hours and then with 50 V for 8 hours. First dimension electrophoresis was performed by Isoelectric focusing (IEF), which was programmed at a gradient mode. It was first focused for 3 hours at the different voltages including 500, 1000 and 8000 V, respectively, then continued at 8000 V and finally increased to 50 KVh. The focused strips were equilibrated in buffer with 6 M urea, 50 mM Tris–HCl, 30% glycerol, 2% SDS and trace bromophenol blue, and were subsequently treated by the reduction of DTT and alkylation of iodoacetamide. The treated strips were transferred into 12% uniform SDS poly acryl amide gels (second dimension of electrophoresis) running in 2.5 W each gel for 30 min and 15 W each gel until the bromophenol blue dye reached the bottom of the gel. The gels were visualized by Coomassie brilliant blue staining and scanned by BioRad Image Scanner. Finally, protein expression alteration analysis was performed by Same Spots software based on above significant score threshold (Fold>2, p ≤0.05). Proteins were subjected to MALDI-TOF mass spectrometer and were identified by Mascot search using the peptide mass finger printing data. 

## Results

**Protein profile of AGS cells upon infection with** ***H.*** ***pylori***

To better understand the molecular mechanisms and the proteins differentially expressed in infected host, human gastric epithelial AGS cells were infected by an Iranian *H.Pylori* isolate. The altered protein patterns separated by 2-DE using pH gradients of 3-10nl were scanned by BioRad Image Scanner and after expression analyzing two spots were identiﬁed by MALDI-TOF MS analysis. 

**Figure 1:**
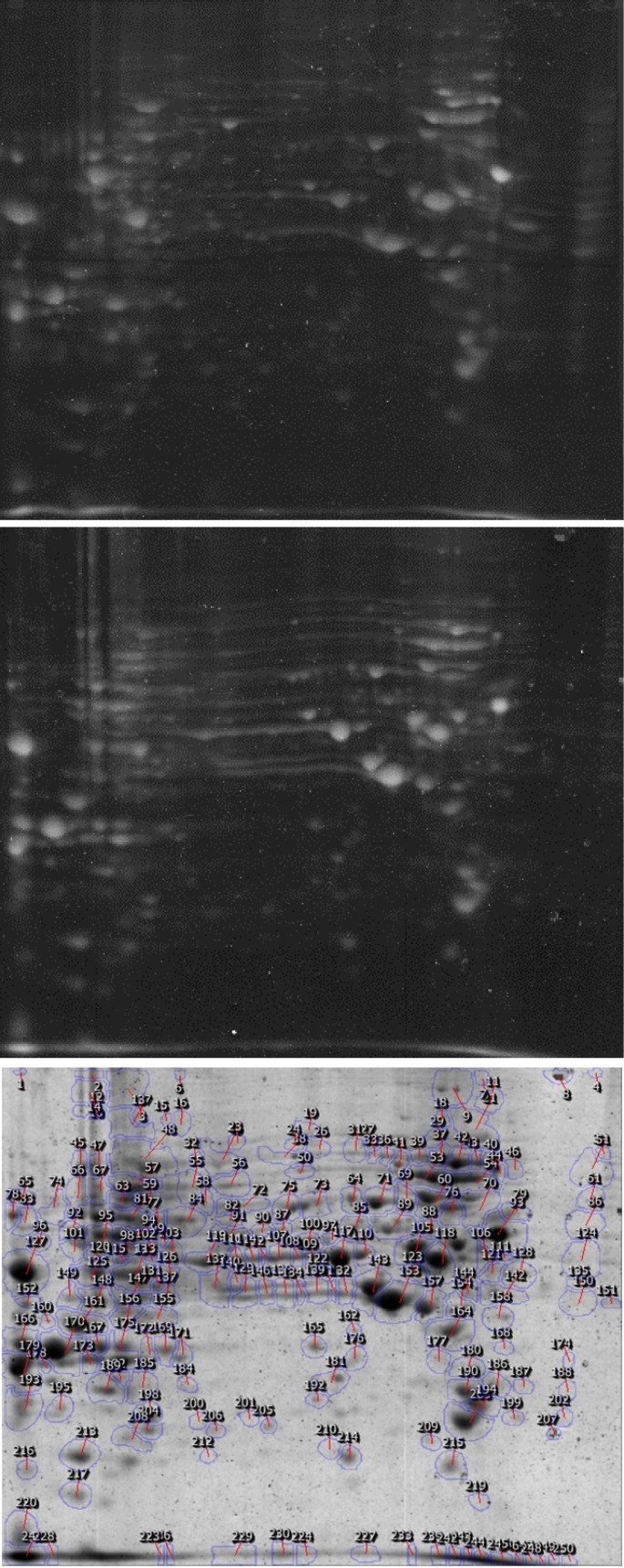
2-DE gel map derived from non-infected and H. *pylori* infected cells (a) Protein profiling of the AGS infected cell with an Iranian *H.Pylori* isolate (b) Protein profiling of the AGS uninfected cell. (c)The gel of uninfected cells was selected as reference gel and the spots were marked on this gel

**Figure 2:**
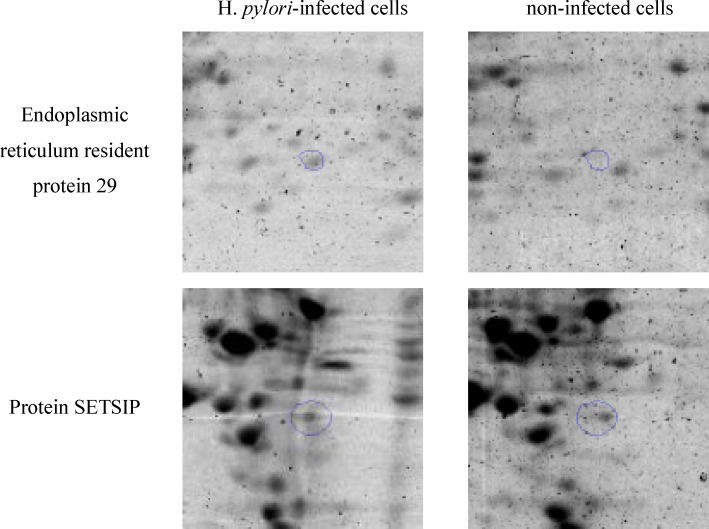
Segments of the 2-DE gel map derived from non-infected cells and *H. pylori*-infected cells. These 2 spots of increased intensity from infected samples as compared to non-infected samples were selected to be identified by MALDI-TOF MS analysis

Figure 1 A shows 2-DE gel of uninfected AGS control cell and Figure 1B 2-DE shows analysis of total proteins from AGS infected with *H.* *pylori*. The gel of the uninfected cells was selected as reference gel and the spots were marked on this gel as in the Figure 1C. Fig. 2 shows segments of the 2-DE gel map derived from non-infected cells and *H.* *pylori*-infected cells. 

Among the approximately 250 protein spots resolved on 2-DE gel, with considering threshold (Fold>2, p ≤0.05), 40 spots of significantly different intensities between the 2-DE gels from the non-infected and infected AGS samples were detected. 

Altogether, 2 spots of increased intensity from infected samples as compared with non-infected samples were selected for next steps. These spots were excised from 2-DE gels and subjected to MALDI-TOF identification assessed to be significant by Mascot factor. Mascot search using the peptide mass ﬁnger printing data indicated that these two up-regulated proteins in *H*. *pylori*-infected cells were Protein SETSIP and Endoplasmic reticulum resident protein 29 (Table 1).

**Functional roles of the up-regulated proteins induced by** ***H.*** ***pylori* infection in AGS**

 Proteomic analysis results showed that the differentially expressed proteins by *H.* *pylori* infection are related to the mechanisms to deal with stress conditions and transcriptional activator involved in cell reprogramming.

## Discussion

*H. pylori *alters protein expression in infected cells. In the present study, we used 2-DE/MS analyses to characterize the response of AGS to *H.* *pylori* infection. The findings of this study indicated that differentially expressed AGS proteins by an Iranian *H. pylori* isolate were related to the mechanisms to deal with stress conditions and transcriptional activator involved in cell reprogramming. The results of the other studies indicated that the differentially expressed proteins suggest the possible involvement of motogenic response, cell morphology, cytoskeletal rearrangements, apoptosis, proliferation pattern and cytokine induction pathways by *H.* *pylori* infection (9, 10).

SETSIP plays a role as a transcriptional activator involved in the early stage of somatic cell reprogramming. Studies show that during stimulating pluripotent stem (iPS) cells, from somatic cells, with VEGF, endothelial cell differentiation induced and SETSIP translocated to the cell nucleus where it binds to VE-cadherin gene promoter and increased vascular endothelial cadherin (VE-cadherin) gene expression. It causes endothelial cell (EC) differenced. These data indicate that reprogramming of ECs via SETSIP has a potential clinical function(12).

ERp29 is an ER component that facilitates transport of synthesized secretory proteins from the ER to Golgi (13). ERp29 is highly expressed in numerous tumors and under the stress conditions, such as basal cell carcinoma and lung cancer progression and also cells exposed to radiation, sperm cells undergoing maturation (14-18).

Cells have developed a diversity of mechanisms to deal with stress-induced cell death, such as cell cycle arrest and activation of the DNA repair. Recent studies indicated that ERp29 can function as a tumor suppressive protein, which protects cells from stress by inhibiting cell growth and primary tumor formation and preventing signaling pathways that facilitate Epithelial–mesenchymal transition (EMT) (16, 19, 20). This protein has important roles in cancer cells including the induction of mesenchymal–epithelial transition (MET) and epithelial morphogenesis as well (21, 22). These functions promote the survival of cancer cells and metastasis (23, 24). 

 Current studies show that ERp29 expression in axotomized neurons, protects cells from apoptosis and promotes neuronal regeneration (25). In breast tumors and colon cancer, ERp29 expression associates with metastasis and prognostic risks of cancer. So, in these cases reduce relapse time of disease and short survival time of patients are expected (26).

Dysregulation of the Wnt/β-catenin signaling pathway plays an essential role in gastrointestinal cancers, including colorectal cancer and gastric cancer (27, 28). In the nucleus, β-catenin interacts with family of transcription factors to induce target gene transcription which are related to proliferation, such as Cyclin D1 and c-Myc (24, 27, 29).

Over-expression ERp29 in MDA-MB-231 cells led to translocation of nuclear b-catenin to membrane and causes abolishes its transcription activity (21). Indeed, ERp29 significantly decreased the expression of cyclin D1/D2 [36], indicating an inhibitory effect of ERp29 on this pathway (30). On the other hands, expression of ERp29 in this cell type increased the nuclear expression of TCF3, a transcription factor regulating cancer cell differentiation (30-32).

On the other hands, *H. pylori* infection is another important factor influencing the Wnt/ β-catenin signaling pathway (33-35). Franco et al. reported that CagA-positive *H. pylori* altered β-catenin localization and increased β-catenin nuclear accumulation in gastric epithelial cells AGS so, the Wnt/β-catenin signaling pathway is activated (36, 37). Results of this study show that on one hand, ERp29 increased by H. pylori infection and on the other hand SETSIP, which has a role in cell differentiation, over expressed in this infected cell line. These results showed that there may be other mechanisms which *H.pylori* regulates β-catenin and the Wnt/β-catenin signaling pathway in AGS cell line.

To understandpathogenic mechanism of *H.* *pylori* infection in the infected host, realizing the role of each protein and its possible relationship with gastric diseases is vital. Since there is a broad biological function of ERp29 in gastric epithelial cells, targeting this protein and/or its downstream molecules as a new anti-cancer molecular therapeutic approach need further studies. 

## Conflict of interests

The authors declare that they have no conflict of interest.
